# Discriminations in medicine and public health: Moral challenges for modern Japanese physicians

**DOI:** 10.1002/jgf2.236

**Published:** 2019-01-17

**Authors:** Yasuharu Tokuda

**Affiliations:** ^1^ Muribushi Okinawa for Teaching Hospitals Okinawa Japan

In recent years, social discriminations against disadvantaged minority people in the U.S. healthcare system and public health are rising, and Dr. Donald M. Berwick, president emeritus at the Institute for Healthcare Improvement, claims in his opinion paper that professional silence in the face of social injustice is wrong and to try to avoid the political fray through silence is impossible, because silence is now political.^1^ Japan now has issues indicating other serious social discriminations that have come out recently. However, Japan does not hear any voice from physician's groups about the ongoing social injustices.

Women have been discriminated unfairly and secretly at entrance into many medical schools in Japan. Several medical schools in Japan recently confessed that they had engaged in systematic discriminatory penalty of test scoring in entrance examinations against women applicants and those who took the test for several times over the past several years. The applicants did not know the discriminatory practices, since admission policy of these medical schools did not describe such practices.^2^ It is well known that there are very few women professors in Japanese medical schools and very few women hospital directors in large hospitals throughout Japan.

Women physicians have been discriminated by way of sexual harassment in Japanese hospitals. Our recent multiple‐choice scenario survey on Japanese physicians also demonstrated that many Japanese staff physicians were unable to provide an acceptable moral choice in issue of sexual harassment.^3^ Some chose the non‐response (Do nothing), indicating there is problematic silence potentially enhancing the sexual harassment culture.

#3 Foreign young workers have been discriminated and forced to work in harsh condition to become seriously ill. The justice ministry revealed that over 100 foreign people working as part of the technical intern program died over the last 8 years because of accidents, illness, and other reasons.^4^ It is well known that there are very few professors of foreign nationals in Japanese medical schools, even for basic science departments which do not require the fluency of Japanese language. Only an English‐speaking faculty exists usually as a teacher of medical English in each medical school throughout Japan and they are typically not involved in the important curriculum development.

Okinawan people, previously independent ethnic group living in remote islands over the south west of Japan, have been discriminated for suffering from unfairly huge burden of the presence of the US military bases. Recently, the national government of Japan initiated construction work on a new US military base despite strong opposition by local Okinawan people, who have experienced a number of adverse health effects and toxic environmental pollution from the US military bases, including severe aircraft noise, asbestos and other carcinogenic exposure, and disrupted biodiversity. Aside from this, there are social issues like rape and violence by military people against local people. However, few medical professional societies in Japan have provided any comments about this issue, although physicians could understand the seriousness of the adverse effects of the military bases to public health.^5^

Discriminations in medicine and public health throughout Japan are now obvious, but physicians seem still silent about these issues. However, for reducing these apparent social injustices, physicians as healthcare professionals are required to have social accountability to make morally sound choice.

## CONFLICT OF INTEREST

The authors have stated explicitly that there are no conflicts of interest in connection with this article.

## References

[1] Berwick DM . Moral choices for today's physician. JAMA. 2017;318(21):2081–2. 29209725 10.1001/jama.2017.16254

[2] The Japan Times . 109 applicants found to be denied entrance in Tokyo Medical University exam‐rigging. Dec 30, 2018. Available from https://www.japantimes.co.jp/news/2018/12/30/national/109-applicants-found-denied-entrance-tokyo-medical-university-exam-rigging/#.XCnKWrqRXDs

[3] Soshi M , Tokuda Y . Sexual harassment: the most challenging issue of medical professionalism in Japan. J Gen Fam Med. 2018;19(4):118–20. 29998039 10.1002/jgf2.186 PMC6030031

[4] The Japan Times . Japan's Justice Ministry reveals 174 foreign technical interns died between 2010 and 2017. Dec 13, 2018 Available from https://www.japantimes.co.jp/news/2018/12/13/national/justice-ministry-reveals-174-foreign-technical-interns-japan-died-2010-2017/#.XCnRW7qRXDs

[5] Tokuda Y , Barnett PB . Constructing a New U.S. Military Base: A Health Threat to Okinawan People. Environmental JusticeVol. 10, No. 1. Published Online:1 Feb 2017. Available from 10.1089/env.2016.0029

